# Anxiety, Stress-Related Factors, and Blood Pressure in Young Adults

**DOI:** 10.3389/fpsyg.2016.01682

**Published:** 2016-10-28

**Authors:** Nicola Mucci, Gabriele Giorgi, Stefano De Pasquale Ceratti, Javier Fiz-Pérez, Federico Mucci, Giulio Arcangeli

**Affiliations:** ^1^Department of Experimental and Clinical Medicine, University of FlorenceFlorence, Italy; ^2^Department of Psychology, European University of RomeRome, Italy; ^3^Department of Clinical and Experimental Medicine, University of PisaPisa, Italy

**Keywords:** blood pressure, anxiety, work-related stress, students, health care professions, health promotion, workplace, occupational medicine

## Abstract

Hypertension (HT) is a long-term medical condition characterized by persistently elevated blood pressure (BP) in the arterial vessels. Although HT initially is an asymptomatic condition, it chronically evolves into a major risk factor for cardiovascular, cerebrovascular, and renal diseases that, in turn, represent crucial causes of morbidity and mortality in industrialized countries. HT is a complex disorder that is estimated to affect more than a quarter of the world’s adult population. It is classified on the basis of both its pathophysiology (primary and secondary HT) and on the resting BP values (elevated systolic, diastolic, and pulse pressure). It originates from a complicated interaction of genes and several environmental risk factors including aging, smoking, lack of exercise, overweight and obesity, elevated salt intake, stress, depression, and anxiety. Anxiety and depressive disorders are the most commonly diagnosed mental disorders, affecting millions of people each year and impairing every aspect of everyday life, both of them characterized by affective, cognitive, psychomotor, and neurovegetative symptoms. Moreover, work-related stress has been considered as an important risk factor for HT and cardiovascular diseases (CVDs). Although different authors have investigated and suggested possible relations between HT, stress, anxiety, and depression during the last decades, a full understanding of the underlying pathophysiological mechanisms has not been satisfactorily achieved, especially in young adults. The aim of this study was to investigate the impact of anxiety and work-related stress in the development of HT amongst young health care profession students and the possible related consequences of early CVDs.

## Introduction

The blood pressure (BP) is the pressure that the blood flow exerts against the walls of blood vessels. It varies in the different parts of the human body according to the phases of contraction of heart and to the conditions of health, exercise, stress, etc. If the term BP is used without further specification, usually refers by antonomasia to the arterial pressure in the systemic circulation. BP is usually expressed in terms of the systolic BP (SBP, maximum pressure), over diastolic BP (DBP, minimum pressure) (Hodgkinson et al., 2015). It is usually measured at a person’s upper arm and is measured in millimeters of mercury (mmHg) because the traditional device used to measure BP, a sphygmomanometer, used a glass column filled with mercury and calibrated in millimeters. Normal resting BP in an adult is within the range of 100–140 mmHg systolic and 60–90 mmHg diastolic (Mancia et al., 2013).

Moreover, during the last two decades, pulse pressure (PP), defined as the difference between SBP and DBP within a normal range of 30–80 mmHg, has received growing attention as an independent predictor of cardiovascular risk (Tin et al., 2002). Some authors, in a meta-analysis of 2,000 combining studies that previously examinated over 8,000 elderly subjects, demonstrated that the risk of major cardiovascular complications and mortality increased by nearly 20% within an increase of 10 mm Hg in PP (Blacher et al., 2000). On the other hand, studies on young adults are still meager and results often contradictory, therefore motivating the increasing need of researching on these subjects in order to fulfill an adequate prevention of such invalidating diseases (Ritvanen et al., 2003; Riese et al., 2004).

Hypertension (HT) is either defined as a transitory or persistent elevation of arterial BP with, arbitrarily, a systolic measures >160 mmHg or more and diastolic measures >90 mmHg (Wright and Diamond, 2006; Poulter et al., 2015). HT represents a worldwide problem afflicting more than a quarter of the world’s adult population, in both developed (333 million) and in developing countries (639 million) (Santulli, 2013). It represents a major preventable risk factor for premature death and disability and, in particular, a crucial risk factor in the development of cardiovascular diseases (CVDs), such as hypertensive heart disease, coronary artery disease, stroke, aortic aneurysm, peripheral artery disease, cerebrovascular disease, and chronic kidney disease (Jennings and Touyz, 2013; Floyd, 2015).

Coronary heart diseases (CHDs) in men are negligible until the age of 40 years, they emerge between 40 and 50 years and then grow exponentially with age; in women occur at ages 50–60 years and grow rapidly. The disadvantage of men compared to women is more pronounced in young people and tends to decrease with age: the lowest frequency of ischemic heart disease in women than in men is particularly evident in the reproductive age. The prevalence difference between the sexes is accompanied by differences in clinical manifestations: women suffer, in fact, more frequently from sudden death, silent heart attack, and angina pectoris (Maas and Appelman, 2010; Khamis et al., 2016).

Notwithstanding the strategies of screening, prevention, and treatment, the prevalence of HT is increasing throughout the lifespan, along with the increasing of population aging, sedentary life-style and high-calorie food intake (Hansen et al., 2007; Floyd, 2015). Moreover, an appropriate BP control is achieved only by less than a half of patients receiving treatment (Lewington et al., 2002; Falaschetti et al., 2014). Last but not least, the financial burden for EU health systems associated with CVDs has been estimated to be nearly € 110 billion in 2006 (corresponding to 10% of total health care expenditure across EU), and the direct cost associated with HT was estimated to be € 51.3 billion (Mennini et al., 2015).

Although the etiology of HT has been widely studied in depth during the last decades, it still remains far to be completely understood, as it results from a complicated interaction of genetic and several environmental risk factors. Furthermore, the development of HT is associated with several demographic, lifestyle, and psychosocial variables (Pilic et al., 2016). Examples of demographic variables related to CVDs are aging, ethnic group, geographic regions, lower income, lower educational attainment, difficulty of access, and lower quality of public health services (Ferguson et al., 2008; Hosey et al., 2014). As far as lifestyle variables are concerned, robust evidence is available that aging, smoking, alcohol consumption, lack of exercise, disruption of circadian rhythms, diet poor in fruit and vegetables, overweight, obesity [pathological elevation of the body mass index (BMI)] and elevated salt intake are related to an increased risk of HT and any CVDs (Tanuseputro et al., 2003; Pilic et al., 2016).

Other risk factors, such as psychosocial ones and mental disorders, have also been investigated by several authors, nevertheless the relation with HT results less clear and, sometimes, controversial (Kessler et al., 2005; Sparrenberger et al., 2009; Spruill, 2010; Graham and Smith, 2016; Mermerelis et al., 2016; Ventura and Lavie, 2016). However, literature agrees that perceived stress amongst health care profession students is high and that a significant number of them may develop a psychological morbidity (Pryjmachuk and Richards, 2008; Nechita et al., 2014; Wallace et al., 2015).

Work-related stress is involved in the development of HT and a consistent number of studies examined different common work-related aspects including job insecurity, strain and control (job quality), satisfaction, wages, work hours, and perceived dissatisfaction (Baldasseroni et al., 2005; Rau, 2006; Wright et al., 2011; Johansson et al., 2012; Leigh and Du, 2012; James et al., 2013; Modrek and Cullen, 2013; Smith et al., 2013; Ford, 2014). Other research had already shown how stress may cause changes in BP, increased cholesterol blood levels, triglycerides, hematocrit, fibrinogen, and blood fluidity. Mental stress may cause an abnormal activation of the sympathetic nervous system (SNS) triggering hormonal cascades that interfere with BP, increased coagulation and platelet activity, factors that can act as “triggers” of cerebrovascular events (Marazziti et al., 1990; Jönsson et al., 2015; Hirokawa et al., 2016).

A recent review of literature underlined how unemployment, extended work hours, job instability, low wages, job strain, and sleep disorders were linked to an increased risk of HT (Cuffee et al., 2014). Recently, some authors (Trudel et al., 2016) evaluated the effect of repeated job strain and effort–reward imbalance (ERI; Siegrist, 1996) reporting that men chronically exposed to an active job, compared with not exposed men, presented a cumulative incidence of HT over 5 years. On the other hand, women who had experienced onset of ERI exposure were found to have a higher systolic ambulatory BP, in contrast with non-exposed women. Some authors reported a positive significant association between job strain and arterial stiffness in working men, while they did not found the same evidences in women, and others, instead, reported a negative association in a group of healthy male employees (Nordstrom et al., 2001; Hintsanen et al., 2005; Nomura et al., 2005; Bugajska et al., 2008).

Epidemiological studies have repeatedly investigated the association between anxiety and HT, with unclear results. Pan et al. (2015), in a recent systematic review and meta-analysis, summarized the current evidence from cross-sectional and prospective studies and their results suggest that there is an association between anxiety and increased risk of HT. These results support early detection and management of anxiety in hypertensive patients.

Depression is a significant and independent risk factor for HT, especially in young people (Shah et al., 2011; Jackson et al., 2016; Mermerelis et al., 2016). Depression could lower the heart rate variability, a normal feature of the heart rhythm: a too low value indicates a greater chance of dying as a result of CVDs. Again, the psychological condition may worsen the inflammatory response or raise the levels of blood cortisol (Shah et al., 2011). Amongst women its role seems to be even pair to traditional factors such as smoking, obesity, diabetes, and HT. Not surprisingly, Shah et al. (2011) observed that women with a history of suicide attempts present a greatest risk of dying from CVDs, three times more than the other (for males was 2.4 times higher than the other). Moreover, the authors have highlighted that these women have a 14 times greater risk of dying from heart attack (men stops to 3.5 times).

Nonetheless the growing body of evidences, the pathophysiological mechanisms underlying the relations between psychosocial factors and elevated BP remains still unclear or even contradictory and needs further investigation, especially in young people. Indeed, adolescents and young adults constitute a population with specific characteristics, which must be taken into account in the management of occupational risks (Caricati et al., 2016). In this sense, considering also the lifestyles that young people are taking in recent years, the evaluation of cardiovascular risk at early age is becoming increasingly important (Mangena et al., 2016).

Effort–rewarding balance could also explain the phenomenon of over identification in special groups of young people, such as health care profession students. In fact, these students experience—in addition to a repeated job strain—a continuous ERI for the whole course of studies due not only to the specific characteristics of a health care profession but, moreover, to the difficulty in entering into employment and in the precariousness of employment contracts. These problems may be even more evident in the current economic crisis (Van Hal, 2015).

Psychological wellbeing has consistently been shown to be related to job satisfaction (Wright et al., 2007; Merino and Privado, 2015). Melamed et al. (2001) have hypothesized that job complexity affects changes in BP levels and job satisfaction over 2–4 years of follow-up. Accordingly, job dissatisfaction has been reported to be an important and powerful source of psychological strain, in particular associated to elevated BP in women with low levels of social support (Ford, 2014).

In particular, chronic exposure to stressors would trigger a vicious cycle consisting in changes of emotional set points. These changes would bolster chronic readiness for future stressors and, finally, culminate in chronically altered affective states and perceived stress. SNS is also involved, as emotional accommodations are actually able to affect its set points especially in shifting basal physiological arousal and altering resting or baseline BP levels (Ganzel et al., 2010; Ganzel and Morris, 2011; Ford, 2014). It has long been known that perceived dissatisfaction at work should be considered a relevant chronic stressor, directly involved either on mental and on physical health, especially BP (Lindgärde et al., 1987; Kivimäki et al., 2008; Virtanen et al., 2013). For these reasons, it has been proposed to check periodically the pulse of employees in order to perform a simple yet accurate screening of heart disease risk, specifically focusing their attention on working population of 50 years old and older (Mathile, 2013). Amongst this age-range, Wright et al. (2007) report that BP differences of 60 mmHg (or more) are more risky for CVDs and that both employees and employers should be aware of the potential problems resulting. On the other hand, there is a lack of studies investigating screening of BP, chronic work stressors and anxiety in young people. Anyhow, studies of this kind may be relatively uneasy to perform due to the enormous number of variables involved, of confounding factors and of the umpteen existing methods (Rosenthal and Alter, 2012).

Therefore, the aim of this paper was to study the role of work-related stress and anxiety in the development of HT in a sample of young health care profession students and the potential consequences of early CVDs. While considering the literature analyzed above, poor student satisfaction and over-identification with University may be considered as stress-related factors. Based on theoretical and empirical arguments, we propose the following three hypotheses:

*Hypothesis 1*: Controlling for differences in gender and age, life style variables, and anxiety and stress-related variables, will be related to systolic pressure in a young population of students.
*Hypothesis 2*: Controlling for differences in gender and age, life style variables, and anxiety and stress-related variables, will be related to diastolic pressure in a young population of students.
*Hypothesis 3*: Controlling for differences in gender and age, life style variables, and anxiety and stress-related variables, will be related to the overall cardiovascular health and PP in a young population of students.

In investigating our hypotheses, we followed the recommendations of leading cardiovascular schools (Frese and Zapf, 1988; Kristensen, 1996; Kasl, 1998) of incorporating objective-based cardiovascular measurements in the study designs. Both SBP and DBP were considered separately since they seem differently related to CHD, and, as an integrated measurement, with PP.

## Materials and Methods

Data were collected between 2015 and 2016. A survey was administered by resident physicians in occupational medicine to a consistent sample of European students of an Italian university. In total, 412 students—belonging to several health care profession courses—participated in the study: 283 participants were women (68.7%) and 129 were men (31.3%). The average age ± standard deviation was 23.9 ± 7.5 years.

Moreover, besides the mandatory health surveillance pursuant to the Italian Legislative Decree no. 81/2008 and subsequent amendments, the occupational physicians of a large Italian University Hospital investigated with particular attention health behaviors and cardiovascular health of the participants.

DBP and SBP readings were obtained by using the same professional aneroid sphygmomanometer on each student’s right arm in a seated position maintained for at least 5 min in a constant room temperature. The instrument, owned by the University Hospital, is regularly calibrated by professional technicians. The diastolic scores ranged between 70 and 90 mmHg; the systolic scores ranged between 100 and 170 mmHg.

Life-style variables were also investigated during the medical consultation. Smoking status was categorized into *non-smoker*, *former smoker*, or *smoker*. Drinking alcohol was categorized into *non-drinker*, occasional *drinker*, *drinker*.

The frequency of physical activity including sport, stretching exercise, and walking was dichotomously categorized into *yes* or *not*. BMI was calculated following the literature recommendation. Students’ height and mass were measured during the medical examination.

### Instruments

#### The Anxiety Scale of the Italian Version of the General Health Questionnaire (GHQ-12)

The Italian version of the scale assesses whether the respondent has recently experienced a particular symptom or behavior. Each item is rated on a 4-point scale (less than usual, no more than usual, rather more than usual, or much more than usual) measuring a perception of psychological distress. A higher score indicates a greater degree of psychological distress (Piccinelli et al., 1993).

#### Student Satisfaction

Overall student satisfaction was assessed on a single item: “How satisfied are you with your present studies?” A meta-analysis, in which single-item measures were correlated with scales measuring overall job satisfaction, found that single-item measures were sufficiently reliable (Wanous et al., 1997). This scale was adapted for the student setting.

#### University Identification

The present study aimed to use a specific model of identification, especially developed for students. We used a single-item graphic scale for the measurement of identification with university adapted by the study of Shamir and Kark (2010). The scale was based on conceiving of identification in terms of distance or overlap between entities in a cognitive space. The content, construct, and predictive validity of this single-item measure have previously been investigated (Shamir and Kark, 2010).

#### Control Variables

Gender and age were included as control variables because they have been identified as possible confounders of the relation between stress-related factors anxiety and BP as explained in the Section “Introduction.”

### Procedure and Data Analysis

Descriptive statistics, Pearson’s *r* correlation and hierarchical regressions were performed on the study data using SPSS version 20 (SPSS Inc., Chicago, IL, USA). We used hierarchical regression as an analytical strategy because it provides statistical tests that allow for predictive conclusions: (1) the power of each block of variables (i.e., demographics, lifestyle variables, and anxiety); (2) the unique relationship between each predictor within each block and the dependent variable; and (3) the predictors with the strongest relationship with the dependent variable across blocks of variables (Midgley and Urdan, 2001; Wolters, 2004).

In order to better highlight the incremental contribution of each psychological variable (anxiety, student satisfaction, and university identification), our analyses proceeded in three blocks; these blocks are shown in **Tables 2**–**4**. In the first block, we added demographic variables (age and gender). In the second block, we added lifestyles variables (smoking status, alcohol consumption, BMI, and sport/exercising). These were followed by psychological variables in the third block.

## Results

Descriptive statistics and the correlations between demographics, anxiety, stress-related factors, and BP are reported in **Table 1**.

**Table 1:** Correlations among the variables.

Variable	1	2	3	4	5	6	7	8	9	10	11	12
1. Gender	–	0.00	-0.14ˆ**	0.02	0.05	0.05	0.02	0.03	0.00	-0.20ˆ**	-0.10ˆ**	-0.22ˆ**
2. Age	0.00	–	-0.03	-0.04	-0.2	0.20ˆ**	-0.02	0.04	-0.10ˆ*	0.02	0.11ˆ*	0.07
3. Exercising	-0.14ˆ**	-0.03	–	-0.02	-0.03	-0.07	0.10ˆ*	0.08	-0.01	-0.05	-0.18ˆ**	-0.13ˆ**
4. Alcohol	0.02	-0.04	-0.02	–	0.02	0.02	0.00	0.04	0.01	0.08	0.07	0.10ˆ*
5. Smoke	0.05	0.02	-0.03	0.02	–	0.03	0.05	0.00	-0.07	-0.09ˆ*	-0.03	-0.09ˆ*
6. Body mass index	0.05	0.19ˆ**	-0.07	0.02	0.03	–	0.04	0.06	0.02	0.03	0.00	0.02
7. Student satisfaction	0.02	-0.02	0.10ˆ*	0.00	0.05	0.04	–	0.36ˆ**	-0.26ˆ**	-0.12ˆ**	-0.03	-0.11ˆ*
8. University identification	0.03	0.04	0.08	0.04	0.00	0.06	0.36ˆ**	–	-0.21ˆ**	-0.01	0.11ˆ*	0.05
9. Anxiety	0.00	-0.10ˆ*	-0.01	0.01	-0.07	0.02	-0.26ˆ**	-0.21ˆ**	–	0.09	0.08	0.11ˆ*
10. Pulse pressure	-0.20ˆ**	0.02	-0.05	0.08	-0.09ˆ*	0.03	-0.12ˆ**	-0.01	0.09	–	0.14ˆ*	0.86ˆ**
11. Diastolic pressure	-0.10ˆ*	0.11ˆ*	-0.18ˆ**	0.07	-0.03	0.00	-0.03	0.11ˆ*	0.08	0.14ˆ**	–	0.63ˆ**
12. Systolic pressure	-0.22ˆ**	0.07	-0.13ˆ**	0.01ˆ*	-0.09ˆ*	0.02	-0.11ˆ*	0.05	0.11ˆ*	0.86ˆ**	0.63ˆ**	–

*^∗^p < 0.05; ^∗∗^p < 0.01.*

**Tables 2**–**4** show the results of the regression analyses. The first analysis was the hierarchical regression with SBP as dependent variable and with demographics in the first block and life style variables and anxiety related stress respectively in the second and third block. In the first block, demographic data account for 4% of variance in DBP, with only gender statistically significant. When lifestyle variables were added in the second block, the model was significant, and this dimensions accounted for increased 4% of variance with smoking status, alcohol consumption, and sport/exercising significant. Finally, when anxiety, student satisfaction, and university identification were added in the third block, the model was significant, and these dimensions accounted for a total of 11% of in variance. The final model pertains the significance of all variables with the only exception of smoking status. In anxiety, student satisfaction, and university identification resulted significant and increased the explained variance of 3% over demographics and lifestyle variables. All the psychological variables were significant in the explanation of the SBP. Interestingly, higher level of identification with university was associated positively with higher SBP.

**Table 2:** Hierarchical regression with SBP as criterion variable.

	SBP		
Predictors	Block 1	Block 2	Block 3
Age	0.07	0.07	0.07
Gender	-0.21ˆ***	-0.23ˆ***	-0.23ˆ***
Alcohol consumption	-	0.10ˆ*	0.10ˆ*
BMI	-	0.01	0.00
Smoking status	-	-0.08ˆ*	-0.07
Sport/exercising	-	-0.16ˆ***	-0.16ˆ***
Anxiety	-	-	0.11ˆ*
Student satisfaction	-	-	-0.12ˆ*
University identification	-	-	0.10ˆ*
*R*^2^	0.04ˆ***	0.08ˆ***	0.11ˆ***
*ΔR*^2^	-	0.04ˆ***	0.03ˆ***

*^∗^p < 0.05; ^∗∗∗^p < 0.001.*

**Table 3:** Hierarchical regression with DBP as criterion variable.

	DBP		
Predictors	Block 1	Block 2	Block 3
Age	0.10ˆ*	0.11ˆ*	0.12ˆ*
Gender	-0.10ˆ*	-0.13ˆ**	-0.13ˆ**
Alcohol consumption	-	0.07	0.06
BMI	-	-0.02	-0.02
Smoking status	-	-0.03	-0.03
Sport/exercising	-	-0.20ˆ***	-0.21ˆ***
Anxiety	-	-	0.11ˆ*
Student satisfaction	-	-	0.02
University identification	-	-	0.15ˆ**
*R*^2^	0.02ˆ**	0.05ˆ***	0.07ˆ***
*ΔR*^2^	-	0.03ˆ***	0.02ˆ***

*^∗^p < 0.05; ^∗∗^p < 0.01; ^∗∗∗^p < 0.001.*

**Table 4:** Hierarchical regression with pulse pressure as criterion variable.

	Pulse pressure		
Predictors	Block 1	Block 2	Block 3
Age	0.10ˆ*	0.01	0.01
Gender	-0.10ˆ*	-0.20ˆ***	-0.21ˆ***
Alcohol consumption	-	0.08	0.08
BMI	-	0.02	0.02
Smoking status	-	-0.09ˆ*	-0.08
Sport/exercising	-	-0.08	-0.07
Anxiety	-	-	0.05
Student satisfaction	-	-	-0.12ˆ*
University identification	-	-	0.06
*R*^2^	0.03ˆ**	0.05ˆ***	0.07ˆ***
*ΔR*^2^	-	0.02ˆ***	0.02ˆ***

*^∗^p < 0.05; ^∗∗^p < 0.01; ^∗∗∗^p < 0.001.*

The second analysis used DBP as dependent variable. Demographics accounted for 2% with the significant role played by gender. Lifestyle variables in the second block added 3% of incremental variance with only sport/exercising significant. The final model in the third block with psychological variables added accounted for a total 7% of variance. University and identification and anxiety resulted significant, whereas student satisfaction not.

Finally, when PP was used as dependent variable, the model was significant, and the considered dimensions accounted for a total of 7% of variance. In the final model only gender and student satisfaction were significant pointing out the peculiar role played by this variables. No lifestyle variables were significant.

## Discussion

The models explain, as expected, a limited portion of the variance since the cardiovascular risk is considerably influenced by age. However, it is interesting to note that in such a young population (average age 23.9 ± 7.5 years old) these variables, in particular the psychological ones, may be related to an increase of the BP.

The present findings add further useful information and insight on the role of psychological stressors (and in particular student satisfaction) in determining young students BP and, therefore, cardiovascular health. These results seem to be in line with current literature (Backé et al., 2011; Hirokawa et al., 2016; Lazaridis et al., 2016), although this study is the first of its kind focusing on a sample of young students (average age 23.9 ± 7.5 years old), unlike the vast majority of the researches on cardiovascular risk and work-related stress. SBP appears to be significantly associated to anxiety and to university identification as well as to alcohol use and to sport/exercise (*hypothesis 1*). University identification and anxiety resulted to be significantly associated to DBP along sport/exercise and gender (*hypothesis 2*). Gender and student satisfaction were significantly associated to PP (*hypothesis 3*). Amongst demographic variables, life styles appear important and, in particular, sport/exercising appear significant (*p* < 0.001) in both the first and the second model.

In fact, the evidence of the several benefits provided by physical activity either on cardiovascular and internal diseases or on psychosocial disorders is well known and exhaustively described in scientific literature. Scott (1960) hypothesized that physical activity would ameliorate anxiety by becoming a sort of distraction from its symptoms and that following a healthy life-style may improve social contacts in patients and, therefore, the feedback from their environment.

Positive effects of physical activity on people who experienced normal or increased anxiety levels have been reported in several randomized trials (Bartley et al., 2013; Wegner et al., 2014; Pedersen and Saltin, 2015). Herring et al. (2010), comparing 40 studies in a meta-analysis, infer that anxiety symptoms in people suffering from chronic illnesses, such as CVDs, can be reduced by physical exercise.

Evidence of clinically relevant lowering of BP in hypertensive subjects engaged in physical training is larger and several meta-analyses are in agreement with the results of beneficial effect of physical activity on resting BP (Fagard and Cornelissen, 2007; Cornelissen et al., 2013; Garcia-Hermoso et al., 2013; Carlson et al., 2014; Huang et al., 2014). Neurohormonal, vascular, and structural adaptation mechanisms seem to be triggered by physical exercise and involved in the lowering effect of BP (Predel et al., 2001). Shah et al. (2016), considering a cohort of more than 4,000 young adults followed for an average of 26.9 years, have highlighted the importance of cardiorespiratory fitness as an independent risk factor for CVDs, and found a reduction of 15% of total mortality and 12% of the first cardiovascular event in three decades for each minute of exercise.

A complete understanding of the underlying nature of the existing relationships amongst job strain, identification, satisfaction, work stress, anxiety, and HT remains still unclear and difficult to comprehend because of the several complicated and confounding factors (Pan et al., 2015; Jackson et al., 2016; Maatouk et al., 2016). At the same time, it represents a challenging opportunity to earlier identify possible risk factors and, possibly, novel intervention strategies especially in the young population, that is in the study of cardiovascular risk.

It is noteworthy that the identification factor with university appeared to be positively related to elevated BP. Although it is difficult to establish an accurate underlying mechanism we may speculate that, possibly, an excessive identification results to be more stressful for the students and eventually expressing itself in a rise of the pressure values. In spite of the fact that several authors have been trying to investigate on the impact of stressful psychosocial work environments on health, focusing in particular on the ERI model (Siegrist, 1996) that combines extrinsic and intrinsic factors in studying work-related health (Birgit et al., 2013; Rasmussen et al., 2016; Siegrist and Li, 2016), available data in students and young people are meager. The concept of social reciprocity is the main core of the ERI model whereby money, esteem, and career opportunities (promotion and job security) become rewards achieved throughout job efforts. According to the ERI model, an elevated job effort combined with a poor reward (lack of reciprocity) triggers consistent negative emotions and stress responses that finally result in adverse long-term effects on health. People who show an excessive engagement and a desire of being in control when trying to cope with exacting work situations are expected to exhibit a high susceptibility to these stress responses (“over-commitment”, OC; Siegrist and Li, 2016). If we hypothesize to apply the ERI model to an excessive identification in university, our finding would result to be in line with the bulk of studies proving the association between OC and elevated SBP over time (Gilbert-Ouimet et al., 2012; Trudel et al., 2013; Xu et al., 2013).

Moreover, over-committed people show elevated levels of atherogenic lipids, glucose, and fibrinogen, increased pro-inflammatory activity, and decreased activity of natural killer cells and suffer from fatigue and insomnia. Weiner (1992) hypothesized the existence of an underlying and chronic arousal of the sympathetic-adrenergic system not equilibrated by restoring recovery processes provided by the parasympathetic nervous system. On the other hand, an individual may independently develop overindulgence with his/her work, in presence of increasing self-imposed demands and inability to regulate his/her work habits. Robinson (1998) interpreted this condition as *workaholism*, pointing out that it becomes problematic when the need to work becomes excessive to the point to create a substantial interference with personal health, happiness, family relationships, and social functioning. Workaholic people develop initially aggressiveness, elevated inner tension, and inability to relax (Marazziti et al., 2014) and, subsequently, either psychiatric symptoms such as anxiety, depression, and irritability, either physical problems such as HT, heart and kidney complications (Elowe, 2010).

It is noteworthy that in our study the only significant variable for PP—in addition to the gender (which is the control anyway)—is the student satisfaction. This variable is crucial to the institutional success: an effective institution has satisfied “customers.” In such a context, the satisfaction of the students may have several implications. First of all, satisfied students are more likely to continue their studies and achieve success positions in academia. This is crucial to improve the financial position and reputation of the University. In addition, a high student satisfaction may be also useful to attract new students, which in turn may increase the University’s reputation (Aherne et al., 2016; Grilo Diogo et al., 2016).

Several theories have been proposed in an effort to better understanding the psychosocial dynamics of student satisfaction. For example, the *happy-productive student theory* (Cotton et al., 2002) suggests that student satisfaction is mediated by psychosocial factors such as coping, stress and well-being. Based on this theory, the authors provided evidence that high levels of psychological distress at university related to lower satisfaction. There is a need to identify and manage the causes of dissatisfaction among students in order to improve their psychological state.

The *investment model* explains the relationship between student satisfaction, dropout, and academic performance. Satisfaction increases when the rewards of study increase (higher grades; Hatcher et al., 1992). The model allows an early identification of students at risk of dropout, as well as to implement specific interventions with proper counseling and support measures.

There is also a third approach, which is based on the *theory of consumer satisfaction* (Churchill and Surprenant, 1982). It considers the satisfaction as a function of the extent to which the expectations of the students are positively confirmed during their course of study.

Some limitations of our study should be acknowledged. First of all, it cannot prove causality since its design was cross-sectional. Consequently, the use of a longitudinal design is suggested. A second limitation is the use of some *single-item* research tools; similarly, the stress construct could be better defined (e.g., workload, support of the leaders, etc.). However, we are of the opinion that the collection and the processing of data were easy and more accessible. A third limitation concerns the sample that is not representative of the population of the Italian university students. In fact, our sample consisted of students from one university only, albeit from several health care profession degree courses. A fourth limitation relates to student satisfaction, which is not strictly a stress factor. However, different studies showed how it is an important determinant of a state of wellbeing (Warr, 1990; Faragher et al., 2005; Schéle et al., 2012; Alexopoulos et al., 2014). A fifth limitation is the single-time and manual measurement of BP. A dynamic monitoring of BP in the 24 h would have provided more reliable results but, at the same time, would have reduced students’ participation in the research, because it is a method not widely accepted. Finally, the explained variance, as already explained above, is low. However, since the pressure values are usually low in people in the age range of our sample, we were expecting an even lower explained variance. Moreover, we observed that other studies, in which the parameter of BP was evaluated in combination with stress (e.g., Ford, 2014), did not report high associations also.

The present study definitely deserves follow-up to reach its assigned purposes, in particular by widening the sample and by extending the research to other university students. It would be interesting to evaluate also the consumption of caffeine from the students. On this topic the research perspectives are interesting. For example, a recent Italian study (Palatini et al., 2016)—which involved more than 1,200 patients over 12 years—showed that the heavy coffee drinkers show a risk of cardiovascular events (especially myocardial infarction) four times higher than non-drinkers, while moderate coffee drinkers they have a tripled risk. These effects could be mediated by the long-term influence of caffeine on BP and glucose metabolism (Palatini et al., 2016). However, coffee is not the only source of intake of caffeine. Especially in young people it is necessary to carefully evaluate the consumption of sugary drinks containing caffeine and, in particular, high caffeine content drinks (*energy drinks*), whose sales have increased worldwide over the past decade (Harris and Munsell, 2015; Brothers et al., 2016; Katz, 2016). Finally, it may need to consider other psychological variables, such as emotional intelligence (Di Fabio, 2014, 2015). Recently, Cabello and Fernández-Berrocal (2015) found that women and young adults in general were more likely to be incremental theorists than men and older adults. Furthermore, they found that emotion and emotional intelligence theories mediated the relationship of gender and age with ability emotional intelligence.

We are confident that our results, confirming the three innovative hypotheses, may be profitably transferred within the University organizations not only as an instrument of risk assessment—mandatory under the laws of EU countries (e.g., the Italian Legislative Decree n. 81/2008 and subsequent amendments)—but also, and moreover, to improve their institutional success. With this in mind, we believe that universities should adopt strategies encompassing the consultation of occupational physicians and industrial psychologists, aimed at assessing the satisfaction of health care profession students and at promoting their health. For the first aspect it may be useful, for example, providing constantly updated information about the professional opportunities of each study profile, encourage meetings with the world of work (especially, but not only, with the University hospitals), predispose strategies of stress management training and organize interventions for the prevention of dropouts. As far as the second aspect is concerned, the data collected during the health surveillance of students (such as BP) are a first, valuable, source for developing health promotion programs in both group and individual levels.

## Author Contributions

NM, GG, SDPC, JF-P, FM, and GA equally contributed to all the following issues of the research: conception and design of the work; acquisition, analysis, or interpretation of data for the work; drafting the work and critically revising it; final approval of the version to be published; agreement to be accountable for all aspects of the work in ensuring that questions related to the accuracy or integrity of any part of the work are appropriately investigated and resolved.

## Conflict of Interest Statement

The authors declare that the research was conducted in the absence of any commercial or financial relationships that could be construed as a potential conflict of interest.
